# Statistical Study of the Seasonal Variations in TEC Depletion and the ROTI during 2013–2019 over Hong Kong

**DOI:** 10.3390/s20216200

**Published:** 2020-10-30

**Authors:** Qiang Li, Yanbo Zhu, Kun Fang, Jisi Fang

**Affiliations:** 1 School of Electronics and Information Engineering, Beihang University, Beijing 100191, China; leeqjl@163.com (Q.L.); zyb@adcc.com.cn (Y.Z.); 2 Aviation Data Communication Corporation, Beijing 100191, China; fangjisi@163.com

**Keywords:** TEC depletion, plasma bubble occurrence, EPB duration, seasonal variation, seasonal asymmetry, ROTI

## Abstract

Equatorial plasma bubbles (EPBs) can cause large total electron content (TEC) gradient magnitudes and significant density irregularities. In this paper, depletions and irregularities due to EPBs are identified by using the Global Positioning System (GPS)-TEC time series extracted from nine Global Navigation Satellite System (GNSS) stations over Hong Kong near the equatorial ionization anomaly (EIA) crest region from 2013 to 2019. The correlation analyses between the daily variation in the rate of TEC change index (ROTI) and that of the EPB occurrence rate, depth, and duration are presented. The monthly EPB occurrence rate, depth, duration, and ROTI show strong seasonal variations, with maxima during equinoctial seasons, especially during the moderate-to-high solar activity years of 2013–2016. Furthermore, two seasonal asymmetries can be clearly seen for these parameters from 2013 to 2016. The EPB occurrences rate, depth, and duration vary annually with the solar radio flux at 10.7 cm (F10.7) index. The correlation analyses of the EPB occurrence rate, depth, and duration are found to be much more strongly correlated with the F10.7 index on an annual basis than on a monthly basis. The correlation analysis of monthly variations shows the impacts of solar activity on EPB occurrence, depth, and duration are seasonally dependent, which is significantly greater in the equinoctial seasons and summer than in winter.

## 1. Introduction

Equatorial plasma bubbles (EPBs) refer to plasma depletions frequently observed in the evening and are the major sources of electron density irregularities in equatorial and low-latitude regions [1,2]. The plasma density irregularities within the bubbles can scatter and diffract radio waves, leading to rapid fluctuations in the signal amplitude and phase in a received electromagnetic wave [3]. The significant magnitude of total electron content (TEC) gradients associated with the side walls of bubbles or with plasma density irregularities could potentially cause very large Global Navigation Satellite System (GNSS) positioning errors [4,5].

Physically, the occurrence of an EPB largely depends on the mechanism for producing initial density perturbations and the conditions affecting the Rayleigh–Taylor (R-T) growth rate [6]. Through the R-T instability mechanism acting on the steep density gradient of the F region bottom side, equatorial spread F (ESF) develops shortly after sunset and propagates both upward and toward off-equatorial latitudes [7,8,9]. As the lower-density ionosphere moves upward into the higher-density topside ionosphere, a plasma bubble is created. Most ionospheric density irregularities are created by velocity shear mixing within the bubble gradients along with the formation of drift waves [1,3]. The irregularities start to decay first with the shortest scale length as the night progresses, and then they disappear when solar ionization refills the flux tube at sunrise [3].

Nighttime equatorial irregularities have been observed in ionograms as ESF for a long time in history [1,9]. The EPB/ESF occurrence was found to be dependent on the solar cycle, season, local time (LT), and longitudinal sector based on many studies using observational techniques such as ionosondes [8], very-high-frequency (VHF) radar systems [10], and in situ satellites [11]. Since EPBs cause depletions and irregularities in total electron content (TEC) time series when satellite-to-receiver ray paths pass through the region of plasma bubbles, studies of EPB occurrence based on TEC or rate of TEC change index (ROTI) have also been conducted in recent years [12].

Previous studies have demonstrated that the easily accessible ROTI is an appropriate indicator to describe the strength of ionospheric irregularities [13]. Monthly, seasonal, semiannual, and latitudinal variations in EPB occurrence based on the Global Positioning System (GPS) ROTI over five global regions between 2000 and 2006 were analyzed [14]. The regional diurnal and seasonal variability in the GPS-TEC depletions over South America in 2008 were studied [15]. The occurrence rate, time duration, and depth of TEC depletions and their dependence on the LT and season from 2002 to 2014 were analyzed at the global scale [16]. The characteristics and causes of seasonal variations in EPB over Hong Kong from 2014 to 2017 were studied [17].

Through previous studies, the characteristics of EPB occurrence were observed to be seasonally and longitudinally dependent [14,18]. Based on ROTI observations, Nishioka [14] showed that the two maxima were seen in the annual occurrence variations in African, Asian, and Atlantic regions. Furthermore, the maxima of occurrence rates in the Asian region were in March/April and September/October. The analysis of EPB occurrences over Hong Kong found a maximum during equinoctial months and a minimum during December from 2001 to 2012, except during the solar minimum in 2007–2009 [19]. Although a geometric relationship between the geomagnetic field line and the sunset terminator line was proposed to successfully explain the observed maxima in the occurrence rates during spring and autumn [18], two asymmetries of EPB occurrences observed in previous studies reveal that other factors need to be studied [14,17,19].

An EPB could affect different aspects of received GPS signals. In addition to temporal and spatial variations in EPB occurrences based on solitary observations, potential correlations among multiple observations, such as scintillation indices, ROTIs, and TEC depletions, have been studied. Occurrences of deep GPS-TEC depletions over China’s low-latitude region during the equinoctial seasons from 2011 to 2012 were found to coincide qualitatively with the scintillation index and ROTI [20]. The correlation coefficients between the ROTIs and scintillation indices over Hong Kong from 2012 to 2013 were found to be 0.6 if the data from all GPS satellites were used together [21]. The statistical relationship between the intensity scintillation index S4 and the ROTI was analyzed over the Indian equatorial region [22].

Correlation studies are appealing not only because the very large number of conventional geodetic receivers provides much more data coverage for research opportunities than the limited number of ionospheric scintillation monitoring receivers (ISMRs) [23] but also because there exists a straightforward relationship between the ROTI, irregularity strength, and intensity scintillation index [24]. For instance, the degree to which ionospheric irregularities produce scintillations is dependent on the frequency of the signal compared to the plasma frequency, propagation geometry, and strength of the irregularities [3,24].

In this paper, depletions and irregularities due to EPBs are identified by using the GPS-TEC time series extracted from nine GNSS stations over the Hong Kong region from 2013 to 2019. The temporal variations in the occurrence rate, depletion depth, duration, and ROTI are analyzed on daily, monthly, and semiannual time scales. The relation between TEC depletion and irregularities is studied by conducting correlation analyses between the ROTI and three characteristic parameters of EPBs: the occurrence rate, depth, and duration. The seasonal and yearly variations in the EPB occurrence rates, depths, and durations and their solar activity dependences are presented. The paper shows that the seasonal dependence and asymmetries are manifested not only in the monthly variations of occurrence rate and ROTI but also in the variations of depth and duration. In addition, EPB occurrence rate, depth, and duration are found to be impacted greatly by solar activity and the impact degree is more significant on an annual than on a monthly basis.

## 2. Data and Methods

### 2.1. Dataset

**Figure 1:**
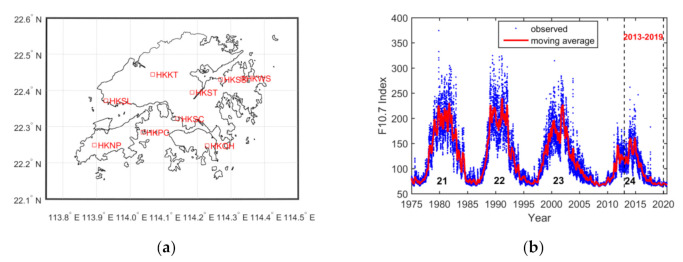
(**a**) Distributions of the nine Global Navigation Satellite System (GNSS) stations in Hong Kong used in the analysis. (**b**) Solar radio flux at 10.7 cm (F10.7) index from 1975 to 2019. The period denoted by two black dashed lines indicates 2013–2019, which is the period studied in the paper.

The paper analyzes the EPBs based on ionospheric slant TEC (STEC) values extracted from GNSS observations. The GNSS observations were recorded from continuously operating reference stations affiliated with the Hong Kong satellite positioning reference station network (SatRef). The distributions of the nine GNSS stations used in the study are shown in Figure 1a. The nine stations were chosen according to the data availability from 2013 to 2019. The station codes, geographic coordinates and geomagnetic latitudes of these stations are shown in Table 1.

Figure 1b shows the solar cycle variations from 1975 to 2020, corresponding to solar cycles (SCs) 21 to 24. The blue dots denote the solar radio flux at 10.7 cm (F10.7) observations, and the red line denotes the 81 day moving averages. The solar activities became weaker from SC 22 to SC 24, the latter of which had the weakest solar activity during the period. The studied period from 2013 to 2019 covers the solar maximum and the solar minimum in SC 24. The solar activity, i.e., the F10.7 index, increased from 2013 to 2014 and then decreased year by year from 2014 to 2019.

### 2.2. Detection Method of Plasma Bubbles

When a GNSS signal propagates through the ionosphere, because of the dispersive nature of the ionosphere the free electrons induce a group delay and a phase advance, which is inversely proportional to the square of the frequency of the signal. This feature could be used to estimate the ionospheric delay by making geometry-free signal combinations from the GNSS dual-frequency signals [25]. Specifically, ionospheric delays from a satellite to a receiver are obtained by differencing the dual-frequency observations between the pseudoranges ($P_{1}$ and $P_{2}$) and between the phases ($L_{1}$ and $L_{2}$) [25,26,27]. The equations are as follows:

(1) $$I_{p}\overset{\mathrm{def}}{=}\frac{P_{2}-P_{1}}{\gamma_{12}}=\mathrm{STEC}+\left(B_{r}+B_{s}+\varepsilon_{2}-\varepsilon_{1}\right)/\gamma_{12}\;$$

(2) $$I_{L}\overset{\mathrm{def}}{=}\frac{\left(L_{1}-L_{2}\right)}{\gamma_{12}}=\mathrm{STEC}+\left(\lambda_{1}N_{1}-\lambda_{2}N_{2}+\upsilon_{1}-\upsilon_{2}\right)/\gamma_{12}$$

(3) $$I_{p1\_L1}\overset{\mathrm{def}}{=}\left(P_{1}-L_{1}\right)/80.6/f_{1}^{2}=\mathrm{STEC}+\;\left(\varepsilon_{1}-\lambda_{1}N_{1}+\upsilon_{1}\right)/\left(80.6/f_{1}^{2}\right)$$

(4) $$\gamma_{12}=40.3/f_{2}^{2}-40.3/f_{1}^{2}$$

where $P_{i}$ and $L_{i}$ are the code and carrier phase measurements, respectively, on frequency $f_{i}$, and $\gamma_{12}$ is a frequency-dependent parameter. $B_{r}$ and $B_{s}$ are unknown hardware biases from the receiver and satellite, respectively, which are relatively stable over several hours to a number of days [28,29]. $N_{i}$ is the carrier phase ambiguity parameter at a wavelength of $\lambda_{i}$. $\varepsilon_{i}$ and $\upsilon_{i}$ are multipath and noise terms of the code and carrier, respectively, on frequency $f_{i}$.

In the above equations, all the ionospheric observables are converted to ionospheric delays expressed in TEC units (TECU), which are convenient for comparing the ionospheric delays from different frequency combinations; 1 TECU is equivalent to 10^16^ electrons/m^2^ column. $I_{p}$ contains the absolute ionospheric delay plus hardware biases. $I_{L}$ and $I_{p1\_L1}$ are impacted by unknown ambiguities in the phase measurement. $I_{p1\_L1}$ relies only on measurements on the first GPS frequency.

For various reasons, a number of cycle slips may occur and lead to sudden discontinuities in carrier phase observations. The carrier phase discontinuity could be detected and repaired by using the method from TurboEdit [26]. Then, to remove effects of unknown phase ambiguities, the leveled carrier-derived ionospheric observable $I_{\phi }$ and leveled code-minus-carrier $I_{\mathrm{cmc}}$ are derived as follows [30,31]:

(5) $$I_{\phi }\overset{\mathrm{def}}{=}I_{L}+\langle I_{p}-I_{L}\rangle {}_{\mathrm{arc}}=\mathrm{STEC}+\left(B_{r}+B_{s}+\langle \varepsilon_{2}-\varepsilon_{1}\rangle {}_{\mathrm{arc}}\right)/\gamma_{12}$$

(6) $$I_{\mathrm{cmc}}\overset{\mathrm{def}}{=}I_{p1\_L1}+\langle I_{p}-I_{p1\_L1}\rangle {}_{\mathrm{arc}}=\mathrm{STEC}+\frac{B_{r}+B_{s}+\langle \varepsilon_{2}-\varepsilon_{1}\rangle {}_{\mathrm{arc}}}{\gamma_{12}}+\frac{\varepsilon_{1}-\langle \varepsilon_{1}\rangle {}_{\mathrm{arc}}}{\left(80.6/f_{1}^{2}\right)}$$

where ${\langle ⋆\rangle }_{\mathrm{arc}}$ means averaging along a continuous arc. The leveled carrier-derived ionospheric observable $I_{\phi }$ has a higher precision than $I_{p}$ and $I_{\mathrm{cmc}}$, and the latter will be used for validation purposes. Although unknown hardware biases are contained in ionospheric observables, they will be canceled out as we are only concerned with the relative value of the STEC in the study. An elevation mask of 15° is used for each satellite and receiver pair.

The temporal rate of ionospheric delay (rate of TEC, ROT) is computed from the difference in the ionospheric delay $I_{\phi }$ divided by the time interval, usually expressed in TECU per minute as follows [12]:

(7) $$\mathrm{ROT}=\frac{I_{\phi }\left(t_{2}\right)-I_{\phi }\left(t_{1}\right)}{t_{2}-t_{1}}$$

The ROTI is usually used to investigate ionospheric irregularities. The ROTI is computed as the standard deviation of the ROT within 5 min windows using the following equation [12]:

(8) $$\mathrm{ROTI}=\sqrt{\langle {\mathrm{ROT}}^{2}\rangle -{\langle \mathrm{ROT}\rangle }^{2}}$$

When the satellite–receiver ray path passes through a plasma bubble, a sudden decrease in the STEC value and then a recovery appears in the GPS-TEC time series. The bubble can be detected using STEC data using the method proposed by Pradipta et al. [32]. In this method, the TEC trend variation is extracted from the original STEC ($I_{\phi }$) time series by using mechanical rotary motion-implicit terrain (MRMIT) filtering [32]. Then, the detrended STEC (dTEC) time series containing the plasma bubble is computed as follows:

(9) $$\mathrm{dTEC}=\mathrm{STEC}-{\mathrm{STEC}}_{f}$$

where ${\mathrm{STEC}}_{f}$ is the TEC trend variation using MRMIT filtering. To identify plasma bubbles, the threshold of minimum TEC depletion is set to 4 TECU. The depletion due to an actual plasma bubble is usually larger than 4 TECU according to previous studies [15,16]. The threshold is chosen to exclude the impact of other disturbances such as traveling ionospheric disturbance.

Three parameters can be identified using the dTEC time series. The depth of an EPB is defined as the maximum magnitude of the TEC depletion derived from the dTEC. The occurrence time of an EPB is defined as the epoch when the dTEC reaches the maximum depletion. The duration of an EPB is computed as the period from the start time of the dTEC decrease to the end time of dTEC recovery.

**Figure 2:**
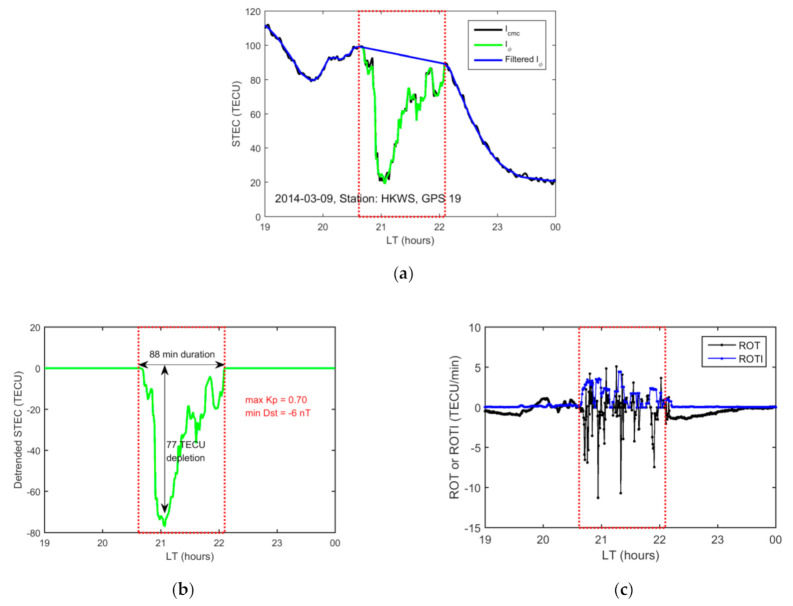
An example of a plasma bubble detected over station HKWS and GPS satellite PRN 19 on 9 March 2014. (**a**) Time series of slant total electron content (STEC) $I_{\mathrm{cmc}}$ (black line), $I_{\mathrm{phi}}$ (green line) and filtered trend using mechanical rotary motion-implicit terrain (MRMIT) filtering (blue line); (**b**) Detrended STEC time series (green line); (**c**) rate of TEC (ROT) (black line) and rate of TEC change index (ROTI) (blue line) time series. The red dashed-line rectangle denotes the period of STEC depletion. The horizontal axis represents the local time (LT, UT + 8).

Figure 2 shows a typical case of an EPB observed by satellite GPS 19 (PRN) from the Hong Kong station HKWS on 9 March 2014 (quiet day with Kp < 1 and Dst ≥ −6 nT). The plasma bubble observed by GPS 19 has a duration of 88 min and a depth of 77 TECU. The large ROTI value corresponds well to the EPB occurrence period, which indicates that severe electron density irregularities inside plasma bubbles cause significant fluctuations in the TEC [14].

## 3. Results

### 3.1. Diurnal Variation in Plasma Bubbles over the HKWS Station

**Figure 3:**
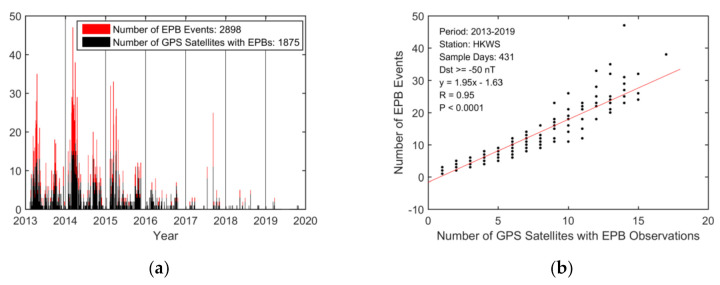
Daily equatorial plasma bubble (EPB) occurrences over HKWS from 2013 to 2019. (**a**) Diurnal variation in the number of EPB events and number of GPS satellites with EPB observations. The total numbers are shown. (**b**) Scatterplot showing the correlation between the daily number of EPB events and number of GPS satellites with EPB observations during a geomagnetically quiet period (Dst ≥ −50 nT). The red solid line is obtained by linear regression. The correlation coefficient (R) and p-value (P) are shown.

The GPS-STEC from station HKWS was analyzed to detect plasma bubbles during the 7 years from 2013 to 2019. As a GPS satellite–receiver ray path can be impacted by plasma bubbles several times during an observation arc, multiple EPB occurrence events can be observed for a GPS-TEC time series. Figure 3 presents the daily variation in EPB occurrence event numbers and the number of GPS satellites with EPB observations. Overall, the daily variations in both numbers showed obvious consistent tendencies from 2013 to 2019. They both vary greatly with solar cycle and season. The correlation coefficient of both numbers was found to be 0.95. Statistical testing for the significance of the relationships resulted in a small *p*-value (*p* < 0.0001) between EPB occurrence event number and the number of GPS satellites with EPB observations, which indicates a strong correlation.

**Figure 4:**
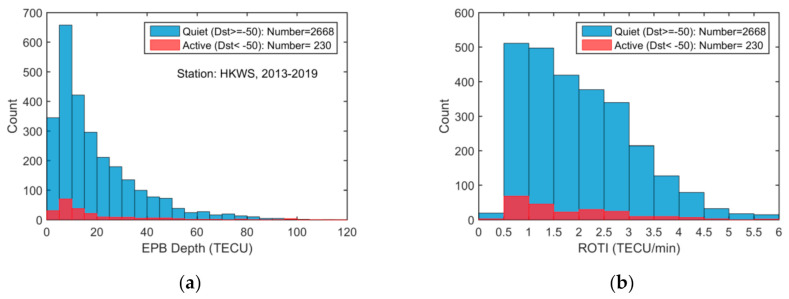

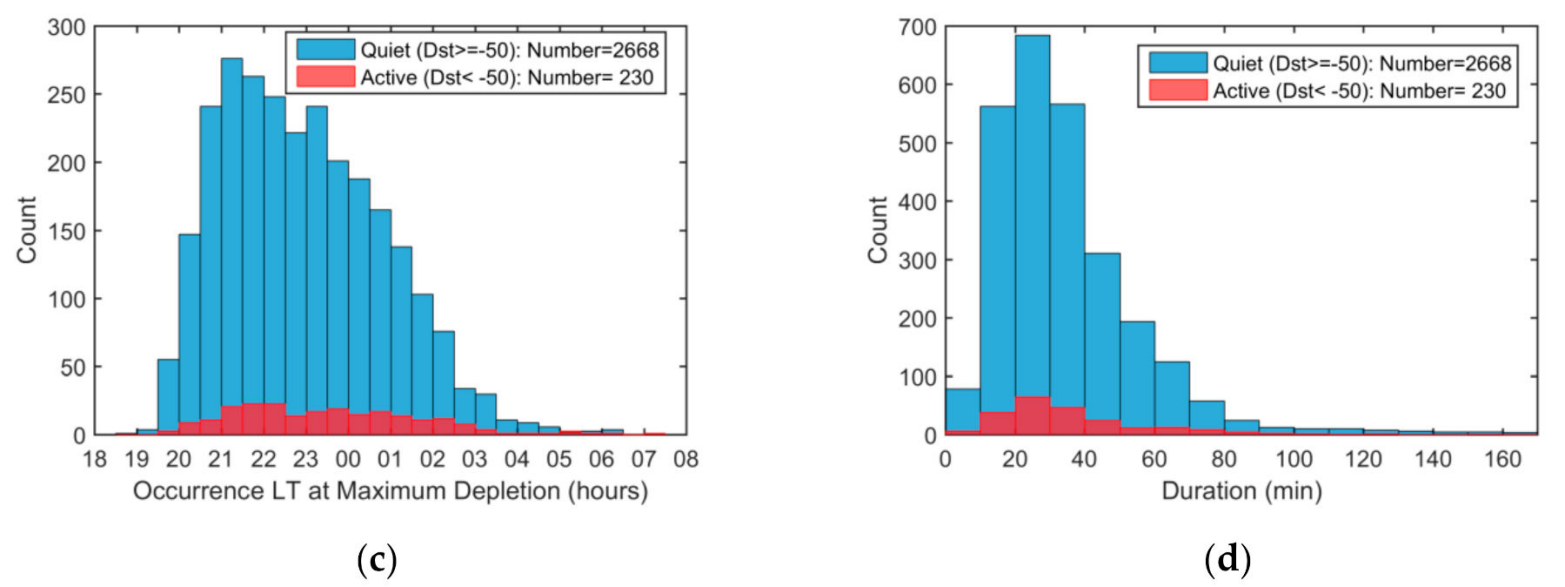
EPB histograms over the HKWS station from 2013 to 2019 of (**a**) the depletion depth in TEC units (TECU); (**b**) ROTI in TECU/min; (**c**) LT at maximum depletion; (**d**) depletion duration in minutes.

In total, 2668 EPB occurrence events were observed on the 431 quiet days and 230 EPB occurrence events were observed on the 39 active days (Dst < −50 nT). Figure 4 presents the observed distribution of the EPB depths, ROTI, LT at maximum depth, and durations from 2013 to 2019. It can be seen that the maximum depth can reach as large as 100 TECU (in the STEC domain), and most of the ROTI values are larger than 0.5 TECU/min, the LTs of the EPB occurrences range from 19:00 to 05:00 LT, and the durations can reach as long as 120 min.

Most EPB occurrences (2668 out of 2898 events) were observed during the quiet period (431 out of 470 days), and furthermore, EPB occurrences can be enhanced or suppressed during the storm period through complicated mechanisms; thus, in the following analysis, only EPBs observed on the geomagnetically quiet days (Dst ≥ −50 nT) were used.

Since we care more about how many satellites would be affected by EPBs, we computed the daily EPB occurrence rate for a particular day using the following equation:

(10) $$daily\;occurrence\;rate\left(\%\right)=\frac{Number\;of\;GPS\;satellites\;with\;EPBs\;on\;the\;day}{Total\;number\;of\;visible\;GPS\;satellites\;on\;the\;day\;}\times 100$$

**Figure 5:**
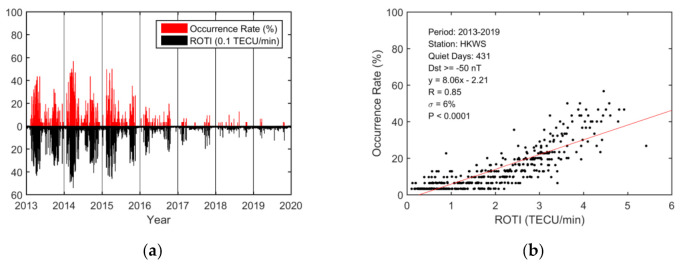
Diurnal occurrence rate over the HKWS station during the geomagnetically quiet period (Dst ≥ −50 nT) from 2013 to 2019. (**a**) Variations in the daily occurrence rate (red bars) and ROTI in 0.1 TECU/min (black bars). (**b**) Correlation between the daily occurrence rate and the ROTI in TECU/min. The red solid line is obtained via linear regression.

We studied the relationship between the variation in the daily occurrence rate based on STEC depletions and the variation in the maximum nighttime ROTI. Figure 5 presents diurnal variations in the daily occurrence rates and ROTI over the HKWS station during 2013–2019. The ROTI values are expressed in units of 0.1 TECU/min for better comparison with the occurrence rate. The occurrence rate and ROTI increased or decreased in a similar manner. The correlation between the daily occurrence rate and the ROTI was found to be strong (R = 0.85).

**Figure 6:**
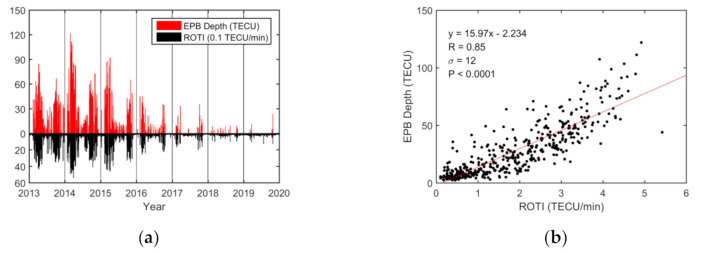
Diurnal EPB depth in TECU over the HKWS station during the geomagnetically quiet period (Dst ≥ −50 nT) from 2013 to 2019. (**a**) Variations in the daily EPB depth (red bars) and ROTI in 0.1 TECU/min (black bars). (**b**) Correlation between the daily EPB depth and the ROTI in TECU/min. The red solid line is obtained via linear regression.

**Figure 7:**
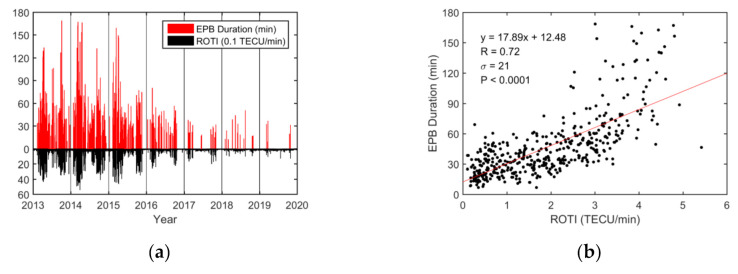
Diurnal EPB duration in minutes over the HKWS station during the geomagnetically quiet period (Dst ≥ −50 nT) from 2013 to 2019. (**a**) Variations in the daily EPB duration (red bars) and ROTI in 0.1 TECU/min (black bars). (**b**) Correlation between the daily EPB duration and the ROTI in TECU/min. The red solid line is obtained via linear regression.

Similarly, the variations in the daily maximum EPB depth and duration were compared with the ROTI. Diurnal variations in the EPB depth and ROTI are presented in Figure 6. The variation in EPB depths was strongly correlated with the ROTI, and the correlation coefficient was 0.85. As shown in Figure 7, the variations in EPB duration and ROTI had a modest correlation coefficient (R = 0.72) and the durations under higher ROTI were slightly more scattered.

### 3.2. Monthly and Seasonal Variation in EPBs

**Figure 8:**
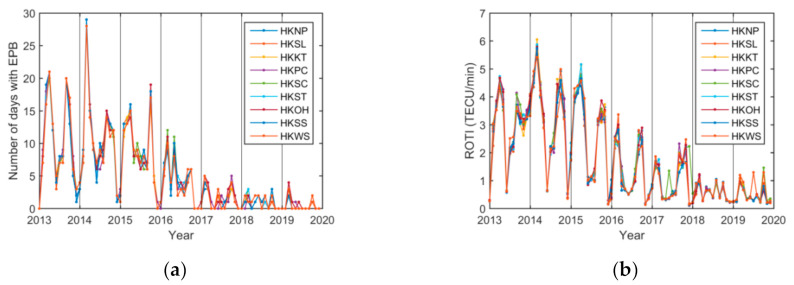
Variation in the monthly EPB occurrences over nine stations from 2013 to 2019. (**a**) Cumulative number of days with EPB occurrences in each month; (**b**) monthly maximum ROTI at nighttime.

Monthly EPB variations from 2013 to 2019 were analyzed for the nine Hong Kong stations. As shown in Figure 8a, the cumulative numbers of days with EPB occurrence in each month for the nine stations were in agreement with each other. The monthly nighttime (from 20:00 to 02:00 LT) ROTI maxima were consistent for the nine stations, as shown in Figure 8b.

To study the monthly and seasonal variations in the EPB occurrences and ROTI, the monthly EPB occurrence rate per station was computed using the following equation:

(11) $$Monthly\;occurrence\;rate\;\left(\%\right)=\frac{Number\;of\;days\;with\;EPBs\;in\;the\;month}{Total\;number\;of\;days\;in\;the\;month\;}\times 100$$

The monthly EPB occurrence rate over the Hong Kong region was then obtained by averaging the monthly EPB occurrence rate per station across the nine stations. Similarly, by averaging the monthly maximum nighttime ROTI per station, the monthly ROTI was derived over the region.

**Figure 9:**
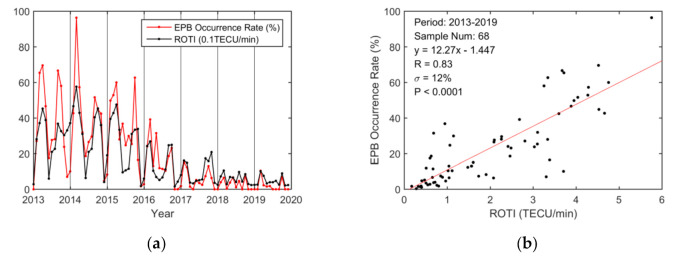
Monthly EPB occurrence rate over Hong Kong from 2013 to 2019. (**a**) Monthly occurrence rate and ROTI in 0.1 TECU/min; (**b**) scatterplot of the monthly EPB occurrence rate vs. the ROTI in TECU/min. The red line is obtained via linear regression.

As shown in Figure 9, there were good correspondences between the EPB occurrence rate and ROTI. The correlation coefficient of the monthly variation was high (R = 0.83), slightly lower than that (R = 0.85) of the daily variation. The values of the occurrence rate and ROTI were obviously larger in the two equinoctial seasons than in the two solstitial seasons during the years from 2013 to 2019 except 2018. During 2018, the difference among the four seasons was not significant. Furthermore, there are two seasonal asymmetries: (1) solstitial asymmetry and (2) equinoctial asymmetry. For the solstitial asymmetry, the occurrence rate was obviously larger in June than in December during 2013–2016. In a similar manner, the ROTI was larger in June than in December during 2014–2016. However, in 2013, the occurrence rate in June was larger than that in December, whereas the ROTI in December was larger than that in June. This phenomenon means that solstitial asymmetry manifested differently in the EPB occurrence rate and ROTI. For the equinoctial asymmetry, both the occurrence rate and ROTI in spring months (March/April) were larger than those in the autumn months (September/October) during 2013–2016.

**Figure 10:**
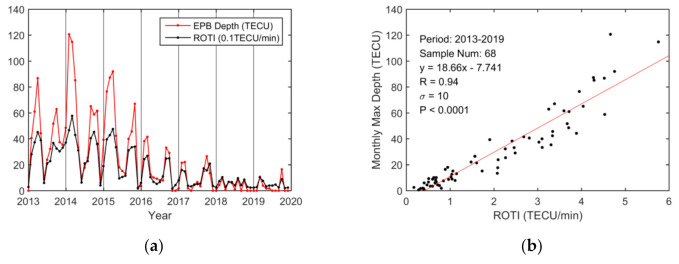
Monthly EPB depth over Hong Kong from 2013 to 2019. (**a**) Monthly EPB depth and ROTI in 0.1 TECU/min; (**b**) scatterplot of the monthly maximum EPB depth vs. the ROTI in TECU/min. The red line is obtained via linear regression.

The monthly variation in the maximum EPB depth was analyzed and compared with the ROTI as shown in Figure 10. Generally, EPB depths showed obvious equinoctial maxima throughout 2013–2019 except in 2018. The EPB depths also presented solstitial and equinoctial asymmetry similar to that observed in the ROTI during 2013–2016. The correlation in the monthly variations between the EPB depths and the ROTI was very high (R = 0.94), which is even larger than that based on diurnal variations (R = 0.85).

**Figure 11:**
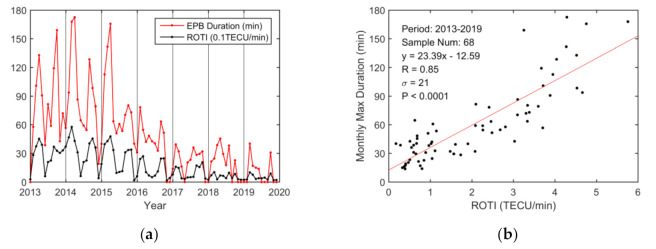
Monthly EPB duration over Hong Kong from 2013 to 2019. (**a**) Monthly EPB duration and ROTI in 0.1 TECU/min; (**b**) Scatterplot of the monthly maximum EPB duration vs. the ROTI in TECU/min. The red line is obtained via linear regression.

The correlation analysis of the monthly variations between the maximum EPB duration and the ROTI is shown in Figure 11. The correlation coefficient is 0.85, which is larger than that based on daily variations (R = 0.72). Two maxima can be seen in the equinoctial seasons from 2013 to 2019. There exists solstitial asymmetry during 2014–2016, whereas there is no obvious difference in 2013. Equinoctial asymmetry similar to that of the ROTI is clearly seen during 2014 to 2016. However, the maximum duration in autumn was larger than that in spring in 2013.

### 3.3. Effect of Solar Activity

**Figure 12:**
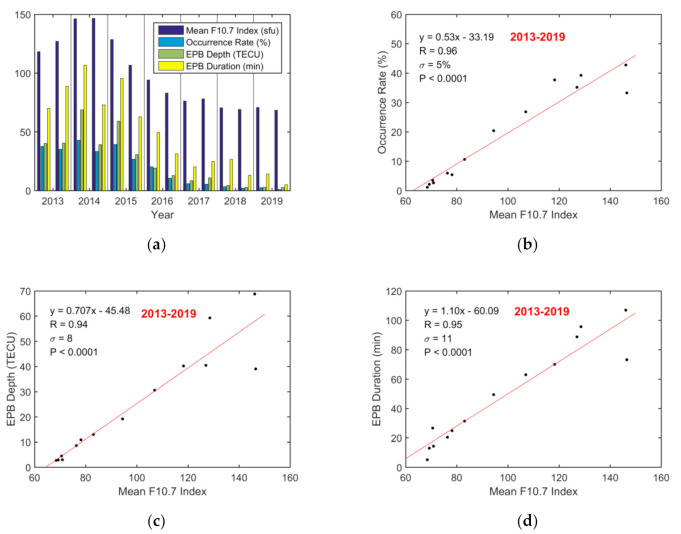
Semiannual variation in EPBs over Hong Kong from 2013 to 2019. (**a**) Semiannual mean F10.7 index, EPB occurrence rate, depth, and duration; (**b**) scatterplot of the semiannual EPB occurrence rate vs. the mean F10.7 index; (**c**) scatterplot of the semiannual EPB depth vs. the mean F10.7 index; (**d**) scatterplot of the semiannual EPB duration vs. the mean F10.7 index. The red lines are obtained via linear regression.

Annual variations in the EPB occurrence rates, depths, and durations were derived for the 7 years from 2013 to 2019 in this study. Figure 12 shows the semiannual variations during the 7 years. The semiannual occurrence rate was derived as the ratio of the number of days with EPB occurrences to the number of observation days in a half year from January to June and from July to December. The semiannual F10.7 index was derived as the average daily value across a half year. The semiannual EPB depth and duration were obtained by averaging the monthly maximum values across a half year.

Figure 12 shows that the semiannual occurrence rate, EPB depth, and duration vary with the mean F10.7 index. A clear SC pattern was observed from 2013 to 2019. In the ascending phase of SC 24 from 2013 to 2014, a small increase was seen in the EPB occurrence rate and depth, whereas a more obvious increase was observed in the EPB duration. These values reached a maximum in the high solar activity year of 2014, and then they decreased gradually from 2014 to the low solar activity year of 2019. Furthermore, the correlation coefficients between the semiannual EPB occurrence rate, depth, and duration and the F10.7 index were 0.96, 0.94, and 0.95, respectively, indicating that they are closely correlated with the F10.7 index on an annual basis.

To study the seasonal impact of solar activity on EPB occurrences, depths, and durations, a correlation analysis was conducted for the monthly occurrence rates grouped into 3 seasons, namely, summer months (May to August), equinoctial months (March, April, September, and October), and winter months (January, February, November, and December).

**Figure 13:**
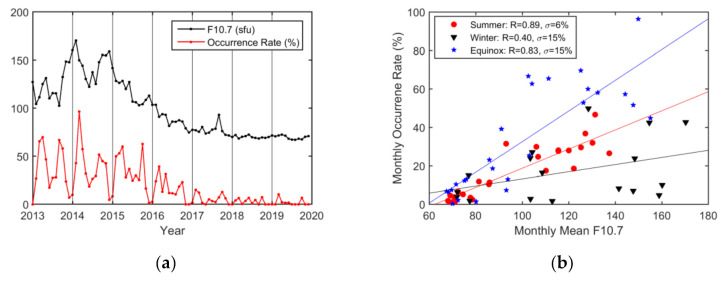
(**a**) Monthly variations in the occurrence rate and F10.7 index; (**b**) scatterplot showing the correlation between the monthly occurrence rate and the mean F10.7 index. The months have been grouped into summer (red circles), winter (black triangles), and equinoctial (blue squares) months. The lines are obtained via linear regression for summer (red line), winter (black line), and the equinoctial seasons (blue line).

The monthly variation in the occurrence rates of EPBs and the monthly mean F10.7 index are shown in Figure 13. A clear seasonal variation was seen in the occurrence rates of EPBs, with the maximum occurrence found in March 2014. The correlation analysis of monthly EPB occurrences against the monthly mean F10.7 index was carried out for the grouped months. The seasonal analysis shows that EPB occurrences were influenced more by solar activity during summer (R = 0.89) and the equinoctial seasons (R = 0.83) than during winter (R = 0.40). Furthermore, the impact of solar activity on EPB occurrences varied more obviously on an annual basis (R = 0.96) than on a monthly basis (R = 0.59).

**Figure 14:**
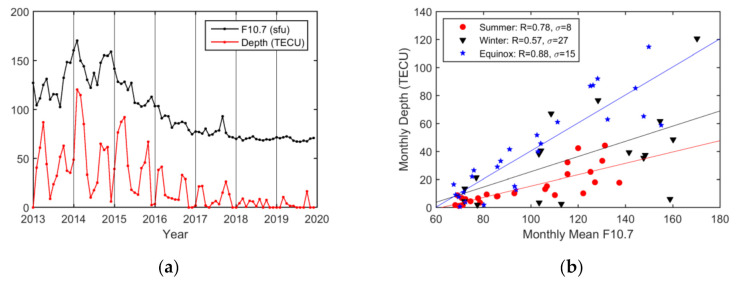
(**a**) Monthly variations in the EPB depth in TECU and the F10.7 index; (**b**) scatterplot showing the correlation between the monthly EPB depth and the mean F10.7 index. The months have been grouped into summer (red circles), winter (black triangles), and equinoctial (blue squares) months. The lines are obtained via linear regression in summer (red line), winter (black line), and equinoctial seasons (blue line).

Similarly, the monthly maximum EPB depths along with the F10.7 index are presented in Figure 14. The maximum depths occurred in February 2014. The seasonal analysis shows that EPB depths were influenced more by solar activity during equinoctial seasons (R = 0.88) and summer (R = 0.78) than during winter (R = 0.57). Furthermore, the impacts of solar activity on EPB depths varied more obviously on an annual (R = 0.94) basis than on a monthly (R = 0.68) basis.

**Figure 15:**
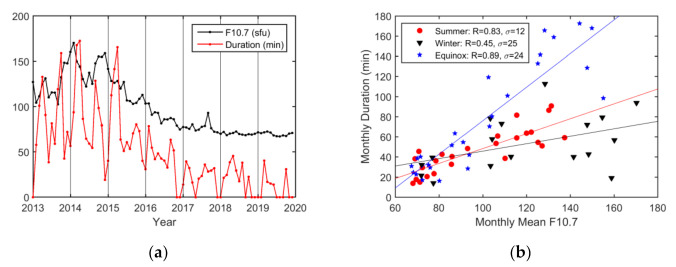
(**a**) Monthly variations in the EPB duration in minutes and the F10.7 index; (**b**) scatterplot showing the correlation between the monthly EPB duration and the mean F10.7 index. The months have been grouped into summer (red circles), winter (black triangles), and equinoctial (blue squares) months. The lines are obtained from the linear regression in summer (red line), winter (black line), and the equinoctial seasons (blue line).

The monthly maximum durations of EPBs along with the F10.7 index are shown in Figure 15. The maximum duration occurred in April 2014. The seasonal analysis shows that EPB durations were influenced more by solar activity during the equinoctial seasons (R = 0.89) and summer (R = 0.83) than during winter (R = 0.45). Furthermore, the impact of solar activity on EPB duration varied more obviously on an annual basis (R = 0.95) than on a monthly basis (R = 0.63).

## 4. Discussion

### 4.1. Diurnal Variation in EPB Occurrence, Depth, and Duration

Based on the analysis from a single station (HKWS) during 2013–2019, the diurnal variations in the EPB occurrences, depths, and durations corresponded well with that of ROTI. They showed similar patterns in maximum and minimum in seasonal scale and solar cycle. On the diurnal scale, the EPB occurrence rate, depth, and duration were highly correlated with the ROTI, with correlation coefficients equal to 0.85, 0.85, and 0.72, respectively.

The magnitude of EPB depth determines the degree of decrease in plasma density of the bubbles. The EPB duration reflects the degree of spatial or temporal scale that a plasma bubble would impact. The ROTI is the metric used to measure the strength of ionospheric irregularity inside or around the region of plasma bubbles with a much larger gradient [5,12]. Ionospheric irregularities occurring inside or around plasma bubbles usually lead to large ROTIs [13]. The relationship between the three EPB parameters and the ROTIs reflects the degree of correlation between electron density depletions and density irregularities. The significant correlation coefficients indicate that greater value in the ROTI is usually closely associated with larger occurrence rate, depth, and duration of EPBs.

### 4.2. Monthly and Seasonal Variation in EPBs

The paper analyzed monthly EPB variations from 2013 to 2019 for the nine Hong Kong stations. The monthly variations in EPBs occurrence rates, depths, and durations were generally consistent with those of the ROTI from 2013 to 2019, and they all showed strong seasonal variations, especially during moderate-to-high solar activity years 2013–2016. The maximum observations in EPB occurrence and ROTI during spring and autumn in this paper were consistent with those from previous reports [14,17,19]. Additionally, the paper showed that the maximum observations in EPB depth and duration also occurred in the spring and autumn.

The correlation coefficients between these parameters and the ROTI were 0.83, 0.94, and 0.85, respectively. Although the correlation coefficient of the monthly variation in EPB occurrences was slightly smaller than that of the daily variation, the correlation coefficients of the monthly variations between the EPB depth and duration and the ROTI were greater than those of the daily variations. This finding indicates that daily variations are more scattered than monthly variations, which might be because subtle variations in various factors that contribute to the nocturnal variations in the EPBs and ROTI are smoothed out during monthly correlation analysis.

Using twenty-three GPS receivers around the magnetic dip equator, Nishioka et at. [14] showed that occurrence rates based on ROTI observations achieved maxima in March/April and September/October in the Asian region. Using GPS-TEC data from 2001 to 2012 over Hong Kong, Kumar et al. [19] reported two clear seasonal maxima in the EPB occurrences corresponding to the spring and autumn months. Tang et al. [17] reported that the seasonal occurrence rate was significantly larger in the two equinoctial seasons than in the solstitial seasons in 2014 and 2015. In the low solar activity year of 2017, the difference in the occurrence rate between summer and the two equinoctial seasons was not obvious. The correlation coefficients between the ROTIs and scintillation indices over Hong Kong from 2012 to 2013 were found to be 0.6 if the data from all GPS satellites were used together [21].

The seasonal variation in plasma bubbles is controlled by the geometry between the geomagnetic field line and the sunset terminator line [18]. In the equinoctial seasons, the E region conductivity is reduced most rapidly due to the closest alignment of the solar terminator with the magnetic meridian, and the rapid reduction is responsible for the enhancement of the vertical plasma drift, which creates a favorable condition for the formation of plasma bubbles.

The paper presented that the seasonal asymmetries were not only manifested in the monthly variation of occurrence rate but also in the variation of ROTI, depth, and duration of EPB during 2014–2019, especially during the moderate-to-high solar activity years of 2013–2016. The patterns of the two seasonal asymmetries were generally consistent with those from previous studies [17]. However, during 2013, the maximum ROTI, depth, and duration were larger in December than in June, and the maximum duration in September was larger than that in March.

The seasonal asymmetries have been studied by previous researchers. Tang et al. [17] reported that the seasonal occurrence rate was greater in spring than in autumn, especially during 2014–2015. Furthermore, Tang et al. [17] observed that seasonal occurrences were more frequent in summer than in winter during 2014 and 2015. Kumar et al. [19] found that the EPB occurrence rate had a higher value in September than in March during the high solar activity year of 2001.

The slight discrepancy between the result in the paper and previous studies indicated complicated mechanisms were involved in interpreting the seasonal asymmetries. The equinoctial asymmetry was assumed to be related to neutral wind in the F region [14] or asymmetry in the electron density distribution [17,18]. The solstitial asymmetry was interpreted by monthly variations in flux-tube-integrated conductivities in the F regions [14] or the seeding mechanism of thunderstorm-driven atmospheric gravity waves [17]. Previous studies [14] also suggested that the discrepancy in the observations of the occurrence rates could be due to the difference in measurement techniques and/or observational periods and/or the location.

Further analysis (not shown here) based on 90 percent instead of the maximum monthly ROTI, occurrence rate, depth, and duration indicates that the values are consistently larger in June than in December during 2013–2016, and they are larger in spring than in autumn. Therefore, the discrepancy in the asymmetries could be attributed to the difference in observation techniques.

### 4.3. Effect of Solar Activity

Annual variations in the EPB occurrence rates, depths, and durations were derived for the 7 years from 2013 to 2019 in this study. The solar cycle dependence was clearly observed from 2013 to 2019. They were highest in the high solar activity year of 2014, and then they decreased gradually from 2014 to the low solar activity year of 2019. The correlation coefficients between the semiannual EPB occurrence rate, depth, and duration and the F10.7 index were found to be 0.96, 0.94, and 0.95. This finding indicates that the EPB occurrence rate, depth, and duration are strongly dependent on solar activity on an annual basis.

Furthermore, the seasonal impacts of solar activity on EPB occurrence, depth, and duration indicated that the impacts of solar activity were most pronounced in the equinoctial months than during winter. Moreover, the differences on impact degree of solar activity were compared respectively between annual and monthly bases for EPB occurrences (annual R = 0.96 vs. monthly R = 0.59), depths (annual R = 0.94 vs. monthly R = 0.68), and durations basis (annual R = 0.95 vs. monthly R = 0.63). The results show that EPBs are influenced more obviously on an annual basis than on a monthly basis.

Tang et al. [17] showed that EPB occurrence from 2014 to 2017 was highest in 2014. Kumar et al. [19] indicated that the EPB occurrence rate varied with solar activity from 2001 to 2012 and reached a maximum during the high solar activity year of 2002. They reported that the correlation coefficient between the annual total number of EPB occurrences and the annual mean F10.7 index was 0.92. Moreover, they observed that the effects of solar activity on EPB occurrences were more pronounced on an annual basis than on a monthly basis. They found that the annual correlation coefficient of 0.92 between the annual number of EPB occurrences and the annual mean F10.7 index was significantly larger than the monthly correlation coefficient of 0.63 between the monthly occurrences and the monthly mean F10.7 index. For the seasonal impacts of solar activity on the number of EPB occurrences, their seasonal analysis showed that EPB occurrences were influenced more during spring and autumn (R = 0.80) than during summer (R = 0.62) and winter (R = 0.68).

The paper shows that the results of solar impact on EPB occurrences are consistent with previous studies. Additionally, the analysis presents that EPB depths and durations are also impacted greatly by solar activity, and the impact degree is more significant on an annual than on a monthly basis. The solar cycle dependence of ionospheric irregularities on the F10.7 index is explained in terms of the enhancement of the pre-reversal electric field amplitude with an increase in the solar UV radiation intensity [33]. The TEC depletion depth and duration characterize different aspects of EPBs, and they are controlled by the same electron dynamical process as the EPBs, and thus are indirectly related to solar activity in a similar manner.

## 5. Conclusions

The paper analyzed the features of plasma bubbles based on ionospheric STEC data extracted from GNSS observations over Hong Kong from 2013 to 2019 covering the solar maximum and the solar minimum in SC 24. By using MRMIT filtering, the detrended STEC time series containing plasma bubbles and related parameters were computed.

The diurnal analysis from a single station (HKWS) showed that the EPB occurrence rate, depth, and duration were highly correlated with the ROTI. The large correlation coefficient between the diurnal variation in the ROTI and that of the occurrence rate (R = 0.85), depth (R = 0.85), and duration (R = 0.72) indicated that TEC depletions and ionospheric irregularities are usually closely associated.

The monthly EPB occurrence rate, depth, duration, and ROTI over the region were derived from 2013 to 2019. They all showed strong seasonal variations, with maxima during equinoctial seasons, especially during the moderate-to-high solar activity years of 2013–2016. The correlation coefficients between these three parameters and the ROTI were 0.83, 0.94, and 0.85, respectively, on a monthly time scale. Although the correlation coefficient of the monthly variation in EPB occurrences was slightly smaller than that of the daily variation, the correlation coefficients between the monthly variations in EPB depths and durations and the ROTI were greater than those of the daily variations. Furthermore, two seasonal asymmetries could be clearly seen for these parameters during the moderate-to-high solar activity period from 2013 to 2016, although they had slightly different patterns, attributed to the difference in observation techniques.

The annual variations in EPB occurrence rate, depth, and duration were presented, which varied with solar activity exhibited by the F10.7 index. In the ascending phase of SC 24 from 2013 to 2014, a small increase was seen, and then they decreased gradually from 2014 to the low solar activity year of 2019. The correlation analysis of the EPB occurrence rate, depth, and duration were found to be more strongly correlated with the F10.7 index on an annual basis than on a monthly basis. The correlation analysis of the monthly variations for three seasonal categories showed that the impacts of solar activity on EPB occurrence, depth, and duration were seasonally dependent, which was obviously more significant in the equinoctial seasons and summer than in winter.

It should be noted that the stations are located near the region at the northern crest of the EIA, therefore the observed features in EPB and ROTI are mostly related to patterns over the specific region. Because of high variability in EPB morphology at the EIA, it is probable that distinct features of EPB may be observed in other different regions.

## Figures and Tables

**Table 1:** List of GNSS stations in Hong Kong with their geographic and geomagnetic coordinates.

Station Code	Geographic Longitude	Geographic Latitude	Geomagnetic Latitude
HKKT	114°03′ E	22°26′ N	12°40′ N
HKNP	113°53′ E	22°14′ N	12°28′ N
HKOH	114°13′ E	22°14′ N	12°29′ N
HKPC	114°02′ E	22°17′ N	12°31′ N
HKSC	114°08′ E	22°19′ N	12°33′ N
HKSL	113°55′ E	22°22′ N	12°54′ N
HKSS	114°16′ E	22°25′ N	12°41′ N
HKST	114°18′ E	22°23′ N	12°38′ N
HKWS	114°20′ E	22°26′ N	12°40′ N
